# Reproducible quantification of cardiac sympathetic innervation using graphical modeling of carbon-11-meta-hydroxyephedrine kinetics with dynamic PET-CT imaging

**DOI:** 10.1186/s13550-018-0421-5

**Published:** 2018-07-20

**Authors:** Tong Wang, Kai Yi Wu, Robert C. Miner, Jennifer M. Renaud, Rob S. B. Beanlands, Robert A. deKemp

**Affiliations:** 1 National Cardiac PET Centre, University of Ottawa Heart Institute, 40 Ruskin St, Ottawa, ON K1Y 4W7 Canada; 2 Department of Physiology, University of Toronto, 1 Kings College Circle, Toronto, ON M5S 1A8 Canada

**Keywords:** Logan, MA1, One tissue compartment, Sympathetic nervous system, HED

## Abstract

**Background:**

Graphical methods of radiotracer kinetic modeling in PET are ideal for parametric imaging and data quality assurance but can suffer from noise bias. This study compared the Logan and Multilinear Analysis-1 (MA1) graphical models to the standard one-tissue-compartment (1TC) model, including correction for partial-volume effects, in dynamic PET-CT studies of myocardial sympathetic innervation in the left ventricle (LV) using [^11^C]HED.

**Methods:**

Test and retest [^11^C]HED PET imaging (47 ± 22 days apart) was performed in 18 subjects with heart failure symptoms. Myocardial tissue volume of distribution (V_T_) was estimated using Logan and MA1 graphical methods and compared to the 1TC standard model values using intraclass correlation (ICC) and Bland-Altman analysis of the non-parametric reproducibility coefficient (NPC).

**Results:**

A modeling start-time of t* = 5 min gave the best fit for both Logan and MA1 (R^2^ = 0.95) methods. Logan slightly underestimated V_T_ relative to 1TC (*p* = 0.002), whereas MA1 did not (*p* = 0.96). Both the MA1 and Logan models exhibited good-to-excellent agreement with the 1TC (MA1-1TC ICC = 0.96; Logan-1TC ICC = 0.93) with no significant differences in NPC between the two comparisons (*p* = 0.92). All methods exhibited good-to-excellent test-retest repeatability with no significant differences in NPC (*p* = 0.57).

**Conclusions:**

Logan and MA1 models exhibited similar agreement and variability compared to the 1TC for modeling of [^11^C]HED kinetics. Using t* = 5 min and partial-volume correction produced accurate estimates of V_T_ as an index of myocardial sympathetic innervation.

**Electronic supplementary material:**

The online version of this article (10.1186/s13550-018-0421-5) contains supplementary material, which is available to authorized users.

## Background

Developed as a positron emission tomography (PET) imaging agent to target the cardiac sympathetic nervous system, carbon-11-labeled meta-hydroxyephedrine ([^11^C]HED) is a norepinephrine analog that is taken up by nerve terminal varicosities in the myocardium, and used to assess sympathetic nerve function [1]. Since its genesis, it has been the cornerstone PET tracer for cardiac sympathetic innervation, employed in determination of neuronal-based defects leading to improved diagnosis and prognosis for pathologies such as heart failure, arrhythmia, and cardiomyopathy, in which cardiac neuronal function is often compromised, leading to decreased catecholamine sensitivity and lowered beta adrenergic receptor density [1]. Using PET [^11^C]HED imaging of cardiac tissues, the volume of distribution (V_T_) of the injected radiotracer is an invaluable metric that quantifies the uptake and retention of tracer, providing an index of sympathetic nerve density and reuptake-1 transporter activity. For cardiac PET applications especially, V_T_ and other kinetic modeling parameters measured in the myocardium may be used to aid in the diagnosis of various innervation and perfusion-based pathologies.

In PET imaging studies, V_T_ is defined as the equilibrium ratio of tracer concentration in tissue to that of unmetabolized parent tracer in plasma, but this direct measurement is typically not feasible due to the long time needed to reach equilibrium. Alternatively, kinetic modeling is commonly used to determine V_T_ from a significantly shorter temporal sample following tracer injection [2]. While the physiological kinetics of [^11^C]HED may be modeled using a two-tissue-compartment model, the one-tissue-compartment (1TC) model has been shown to provide a robust representation with optimal clinical reproducibility in myocardial uptake studies, without sacrificing the accuracy of V_T_ quantification [3]. Two graphical methods reported in the literature for kinetic modeling of reversible-binding tracers are the Logan [4] and Multilinear Analysis-1 (MA1) models [5], which are both computationally simpler than non-graphical (compartmental) methods [6], while being able to provide visual representations of kinetic parameters. The Logan method has been established as the standard graphical model to estimate V_T_ in a wide range of PET applications in the brain and heart, while MA1 was proposed as an alternative numerical formulation to estimate V_T_ with lower noise bias compared to Logan estimates [5]. Although [^11^C]HED is a widely used tracer, a comprehensive evaluation of the performance of the graphical and non-graphical methods to quantify its kinetics has not been performed. Furthermore, the effects of partial-volume losses on quantification of V_T_ have not been well defined in the context of graphical kinetic modeling in the heart, where the effects of blood-pool spillover and motion are more apparent compared to the brain. The goal of this study was to determine a method of partial-volume correction applicable to graphical kinetic modeling and to compare the Logan and MA1 models to the standard 1TC kinetic model for accurate quantification of myocardial sympathetic innervation using dynamic [^11^C]HED PET-CT studies.

## Methods

### Patient study design

Twenty-three heart failure patients were recruited as control subjects for a previous study (PET-OSA: NCT00756366) investigating the effects of continuous positive airway pressure (CPAP) on sympathetic nerve function and cardiac energetics in heart failure patients with obstructive sleep apnea (OSA) [7]. These control patients had the same inclusion and exclusion criteria as the PET-OSA study, except they did not have OSA. Patient demographics were collected at baseline and follow-up visits.

Three of the 23 patients were missing baseline or follow-up PET scans and were excluded. Two other patients were also excluded: one with atrial fibrillation at baseline that was treated before the follow-up scan and the other with uncorrectable severe motion artifact, leaving *N* = 18 subjects included in the final analysis. All patients provided written informed consent, according to the research protocol approved by the Human Research Ethics Board at the University of Ottawa Heart Institute.

### [^11^C]HED PET imaging

[^11^C]HED was synthesized from [^11^C]methyl-iodide and metaraminol-free base, with the use of standard methods for high purity and specific activity [8]. Images were obtained at baseline and follow-up (47 ± 22 days apart) on the ECAT-ART PET (Siemens/CTI, Knoxville, TN) or Discovery RX PET-VCT (GE Healthcare, Waukesha, WI) scanner, with ECG, heart rate, and blood pressure monitored at regular intervals. A transmission scan for attenuation correction was performed using Cs-137 isotope or X-ray CT [9], immediately after which 10–15 mCi (370–550 MBq) of [^11^C]HED was injected over 30 s and a dynamic PET series was acquired over a 40-min period (10 × 10 s; 1 × 60 s; 5 × 100 s; 3 × 180 s; 4 × 300 s) [10]. Image reconstruction was performed using filtered-back-projection with a 12-mm Hann filter and all corrections enabled for quantification of radioactivity concentration [11].

### Tracer kinetic modeling

#### Blood metabolites correction

Quantitative analysis of [^11^C]HED kinetics requires correction for radiolabeled metabolites that accumulate over time in the bloodstream, which are not present in the myocardium [12]. The arterial whole-blood tracer concentration C_WB_(*t*) is typically measured using an image-derived region of interest (ROI) placed in the LV cavity and must be differentiated from the unchanged parent tracer concentration in plasma C_p_(t) as defined using the standardized nomenclature of Innis et al. [13]. The relation between C_p_(*t*) and C_WB_(*t*) was characterized as a time-varying function of plasma-to-whole-blood and unchanged parent-to-metabolized radiotracer in the bloodstream and expressed as a combined parent fraction in plasma pfp(t) function (Additional file 1: Figure S1) derived from studies performed previously in humans [3]:

1 $$ {C}_p(t)={C}_{WB}(t)\times pfp(t) $$

#### Compartment modeling and partial volume correction

The tracer volume of distribution V_T_ in the myocardium is defined as the ratio of the concentration of tracer in tissue divided by the concentration of tracer in arterial plasma, after the system has reached equilibrium (at *t* ≥ T_E_) [13].

2 $$ {V}_T=\frac{C_T\left({T}_E\right)}{C_p\left({T}_E\right)},\mathrm{when}\ \frac{d{C}_T}{dt}=0 $$

Where C_T_ is the concentration of tracer in myocardial tissue and C_p_ is the concentration of tracer in plasma. For a reversible 1TC kinetic model, the rate-of-change of tracer concentration in myocardial tissue is defined using the rate of influx from arterial plasma-to-tissue (*K*_1_) and the rate of efflux from the tissue compartment (*k*_2_) according to Eq. 3.

3 $$ \frac{d{C}_T(t)}{dt}={K}_1{C}_p(t)-{k}_2{C}_T(t) $$

At equilibrium (*t* ≥ T_E_), the rate-of-change of tracer concentration in tissue is equal to zero ($\frac{d{C}_T(t)}{dt}=0\Big)$ [14]. Combining (2) and (3), the volume of distribution may be expressed as:

4 $$ {V}_T=\frac{K_1}{k_2}=\frac{C_T\left({T}_E\right)}{C_{p\left({T}_E\right)}} $$

This simple derivation is applied widely in the analysis of neuro-PET imaging studies. However, in cardiac PET applications, additional image blurring due to cardiac contractile and respiratory motion makes it difficult to deduce the exact boundaries of myocardial tissue based on the measured ROI. There is also a 10–15% fraction of blood volume within normal myocardial tissue that must be considered. These effects may be lessened with modern PET-CT hybrid scanners with improved spatial and contrast resolution, but for cardiac imaging, these effects are still pronounced, necessitating implementation of partial-volume corrections [15, 16]. With partial-volume spillover considered, our model of the imaging process becomes:

5 $$ {C}_{ROI}(t)= RC\times {C}_T(t)+{F}_{WB}\times {C}_{WB}(t) $$

where C_ROI_(*t*) is the measured tracer concentration in the PET myocardial image ROI. F_WB_ is the fraction of whole-blood signal C_WB_(*t*) contained in the measured ROI curve due to imaging spillover effects and anatomical blood volume in the myocardial tissue [11]. C_T_(*t*) is the tracer concentration in the myocardial tissue (excluding blood), and RC is the partial-volume recovery coefficient describing the fractional underestimation of C_T_(*t*) due to limited spatial resolution and myocardial motion blurring. In this study, the value of RC was estimated regionally as 1 − F_WB,_ according to the method of Hutchins et al. [15] used commonly in the compartmental analysis of cardiac PET dynamic imaging studies. Blood spillover from the right ventricle cavity to the interventricular septum was not modeled explicitly. Spillover from the myocardium to blood-pool was not corrected, which might affect C_WB_(*t*) at later time points. Isolating for C_T_(*t*) we have:

6 $$ {C}_T(t)=\frac{C_{ROI}(t)-{F}_{WB}\times {C}_{WB}(t)}{RC} $$

Substituting C_T_ from (5) into the definition of ${V}_T=\frac{C_T\left({T}_E\right)}{C_p\left({T}_E\right)}$ in (1) and assuming *t* ≥ *T*_*E*_, we obtain:

7 $$ \frac{\left[\frac{C_{ROI}\left({T}_E\right)-{F}_{WB}\times {C}_{WB}\left({T}_E\right)}{RC}\right]}{C_p\left({T}_E\right)}={V}_T $$

8 $$ \frac{\left[{C}_{ROI}\left({T}_E\right)-{F}_{WB}\times {C}_{WB}\left({T}_E\right)\right]}{C_p\left({T}_E\right)}={V}_T\times RC $$

C_WB_ may be expressed in terms of C_p_ from (1), only considering *t* ≥ T_E_:

9 $$ \frac{C_{ROI}\left({T}_E\right)-{F}_{WB}\times \left(\frac{C_p\left({T}_E\right)}{pfp\left({T}_E\right)}\right)}{C_p\left({T}_E\right)}={V}_T\times RC $$

Maintaining the same logic as (2) and distinguishing between V_T_ for the volume of distribution that corresponds to the true myocardial tissue compartment (C_T_) and V_ROI_ for the volume of distribution that corresponds to the measured region of interest (C_ROI_), V_ROI_ may be expressed as the ratio: $\frac{C_{ROI}\left({T}_E\right)}{C_p\left({T}_E\right)}={V}_{ROI}$. This can hence be substituted into (9); then, C_p_(t) may also be canceled from the second term of (9), yielding:

10 $$ {V}_{ROI}-\frac{F_{WB}}{pfp\left({T}_E\right)}={V}_T\times RC $$

where pfp(*T*_*E*_) represents the equilibrium value of pfp(*t*). Then, (10) can be rearranged to isolate V_T_ as:

11 $$ {V}_T=\frac{V_{ROI}-\left(\frac{F_{WB}}{pfp\left({T}_E\right)}\right)}{RC} $$

From (11), we propose that V_T_ may be estimated from V_ROI_, with plasma-to-whole-blood and metabolite corrections as well as partial-volume effects considered explicitly.

### Graphical kinetic modeling

The Logan model (12) was derived from the first-order differential equations for general compartment models. Its purpose was to create a graphical method of quantifying V_T_, while making kinetic modeling more mathematically and computationally simple and robust. The base equation, adapted from the original formulation [5] to fit the reversible 1TC model in the context of cardiac PET, is:

12 $$ \frac{\int_0^T{C}_{ROI}(t) dt}{C_{ROI}(T)}=\left({V}_{ROI}\right)\frac{\int_0^T{C}_p(t) dt}{C_{ROI}(T)}+ Int $$

C_ROI_(*t*) and C_p_(*t*) time-activity curves are used as measured input data. At a certain time (t*), the intercept term (Int) will become a constant value [17], at which point the equation becomes a linear system where the slope represents the volume of distribution in the ROI. Since the measured tissue curve C_ROI_(*t*) is subject to blood spillover and partial-volume losses, only V_ROI_ may be obtained from the graphical model directly. Previous applications of this model have estimated V_ROI_ without explicit correction factors for partial-volume effects, which is required for cardiac applications. In our proposed model, Eq. (11) may be used to determine V_T_ from the slope determined by the Logan model.

The second graphical method investigated is the MA1 model, originally formulated as a more numerically stable alternative to the Logan model [6]:

13 $$ {C}_{ROI}(T)=\frac{1}{Int}{\int}_0^T{C}_{ROI}(t) dt-\frac{\left({V}_{ROI}\right)}{\mathrm{I} nt}{\int}_0^T{C}_p(t) dt $$

As with the Logan model, only *T* > t* are used for MA1 analysis. MA1 is a multilinear equation with two independent variables, and the corresponding Logan slope V_ROI_ is equal to the negative ratio of the two coefficients, such that:

14 $$ {V}_{ROI}=-\left(\frac{-\frac{\left({V}_{ROI}\right)}{Int}}{\frac{1}{Int}}\right) $$

V_T_may again be determined from the V_ROI_ value estimated using MA1, according to the relation defined in (11).

#### Determination of t* for graphical models

The estimation start-time (t*) was varied systematically from 1.5 to 20 min for a subset of five [^11^C]HED studies to determine the optimal value to be used for the main analysis. Goodness-of-fit was evaluated on the Logan plot as the Pearson correlation (*r*^2^) of the points from t* to 40 min, indicating the subset of points best described by a line. Since the *r*^2^ is not effective to assess goodness-of-fit of the near-horizontal fitted plane on the MA1 plots, an alternative metric was computed using the relative standard error of the estimate (rSEE) as 1 − SEE/mean. The optimal t* was determined by comparing V_T_ values from the graphical methods to the 1TC model standard. Then, all subsequent analysis was performed using the same start-time for both Logan and MA1 models.

#### PET image analysis

The compartmental and graphical analysis models were implemented in the FlowQuant® analysis software (University of Ottawa Heart Institute, ON). The operator reliability of this automated software has been reported previously [18]. Briefly, the left ventricle (LV) myocardium was segmented automatically and partitioned into voxels using a 2D polar-map representation, with each voxel representing a transmural sub-region of the LV myocardial tissue. The arterial whole-blood (WB) ROI was positioned automatically at the center of the left atrioventricular valve plane. Time-activity curves were generated based on measured tracer activity in the LV cavity C_WB_(*t*) and myocardial tissue C_T_(t) ROIs, as input to the tracer kinetic models.

In each polar-map voxel, the 1TC model rate constants K_1_ and k_2_, as well as V_T_ and the blood spillover fraction F_WB_, were estimated using weighted least-squares regression, according to Eqs. (3), (4), and (5). The Logan and MA1 graphical models in Eqs. (12) and (13) were used to calculate LV polar-maps of V_ROI_. Scan-specific spillover values were calculated as the polar-map median F_WB_ and the corresponding partial-volume recovery coefficient RC (1 − F_WB_), which were then used to estimate V_T_ from the graphical model estimates of V_ROI_ according to Eq. (11). Image and data analyses were performed using MATLAB 2013b (The Mathworks, Natick, MA).

### Statistical analysis

LV median V_T_ values obtained from the 1TC, Logan, and MA1 methods were tabulated. Inter-model and test-retest mean effects were evaluated using two-way repeated measures ANOVA. Bland-Altman analyses and Intra-class correlation (ICC) were employed to evaluate the inter-model (MA1 vs 1TC, and Logan vs 1TC) and test-retest (baseline vs follow-up) reliability [19, 20]. Absolute-agreement ICC with two-way mixed effects was used for the inter-model reproducibility and test-retest repeatability [21]. To correct for skew in the V_T_ distributions, V_T_ values were logarithmically transformed before the ANOVA and ICC analyses. ICC values were categorized as: ICC > 0.90 excellent, > 0.75 very good, > 0.40 good, and ≤ 0.40 poor [22]. The limits-of-agreement of repeated measures were estimated using the following: (i) median difference ± non-parametric repeatability coefficient (NPC = 1.45 × IQR) to account for the variable effect of outliers and (ii) mean difference ± coefficient-of-repeatability (CR = 1.96 × SD). √(3/N) × SD_difference_(*t*_95%, *n* − 1_) was used to calculate the 95% confidence intervals on the limits-of-agreement, where *N* is the number of pairs being analyzed [20]. Differences in V_T_ values were divided by the mean V_T_ to account for the increased variability of differences associated with increased mean V_T_. The NPC was also reported as it is a more robust measure of repeatability [23]. Non-parametric Levene’s test was used to assess the equality of variance between groups. Bias in the Bland-Altman plots was assessed using the one-sample Wilcoxon Signed Ranked test against zero. A 2-tailed *p* value < 0.05 was considered statistically significant for all tests. Statistical analyses were performed using Excel 2016 (Microsoft) and SPSS 20.0 (IBM).

## Results

### Patient demographics

Baseline patient demographics are listed in Table 1. Most patients in this study were male (66.5%) with mean age of 66.5 ± 9.3 years. The majority (83.3%) were classified as having NYHA Class II heart failure and were taking one or more cardiac medications. The demographics were stable at follow-up compared those reported at baseline.

**Table 1:** Patient characteristics (*N* = 18)

Description	Value
Age (years)	66.5 ± 9.3
Body mass index (BMI)	27.5 ± 4.9
Left ventricular ejection fraction (%)	31.8 ± 6.2
Males	12 (67%)
Smoking status	
Current	2 (11%)
Former	8 (44%)
Never	8 (44%)
Diabetes mellitus	6 (33%)
Hypertension	12 (67%)
Dyslipidemia	11 (61%)
Family history of heart disease	8 (44%)
Medications	
ACE inhibitor	16 (89%)
Beta blocker	8 (44%)
Digoxin	6 (33%)
Diuretics	12 (67%)
Statin	13 (72%)
Acetylsalicylic acid	14 (78%)
Plavix	6 (33%)
Coumadin	3 (17%)
New York Heart Association (NYHA)	
Class II	15 (83%)
Class III	3 (17%)
Ischemic cardiomyopathy	
Previous PCI or CABG	13 (72%)
Previous MI	11 (61%)

Values are mean ± standard deviation or number (percent) of patients

### Adjustment of start-time (t*) for graphical models

**Fig. 1:**
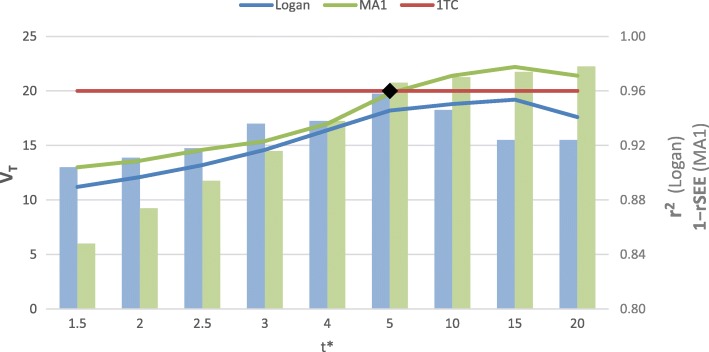
Adjustment of MA1 and Logan model start-time t*, showing the goodness-of-fit metrics for Logan (Pearson *r*^2^) and MA1 (1 − rSEE) as well as the trend of V_T_ values (blue and green lines) for both models compared to the 1TC reference (red line). t* = 5 min produced the highest Logan *r*^2^ with corresponding V_T_ = 18.2 mL/cm^3^, and MA1 V_T_ = 19.8 mL/cm^3^ which was close to the reference 1TC value of 20 mL/cm^3^ (black diamond)

Table 2 shows the goodness-of-fit metrics for the five patients randomly sampled from the entire cohort used for this study. These ranged from 0.80 to 0.99 across all scans and t* values of 1.5–20 min, as summarized in Fig. 1. Corresponding V_T_ values ranged from 9 to 21 mL/cm^3^ for Logan and 10–28 mL/cm^3^ for MA1. The Logan V_T_ increased systematically up to approximately 5–15 min, interpreted as the start of the steady-state (linear) phase. There was no t* value with Logan V_T_ estimates equal to the 1TC reference value (20 mL/cm^3^); therefore, t* = 5 min was selected as the optimal start-time based on agreement of the MA1 V_T_ values with the reference 1TC model. This value of t* also demonstrated the highest Logan *r*^2^ value (0.96), suggesting the best fit of a line was obtained for the points starting at 5 min. MA1 plots exhibited a steady increase in goodness-of-fit up to 5 min (0.97) with relatively little improvement at later start times.

**Table 2:** Effect of graphical modeling start-times (t*) on measured V_T_ and goodness-of-fit values (*N* = 5)

t* (min)	Logan V_T_	Logan *r*^2^	MA1 V_T_	MA1 1 − rSEE
1.5	11.2	0.90	13.0	0.85
2	12.1	0.91	13.6	0.87
2.5	13.2	0.92	14.6	0.89
3	14.6	0.94	15.4	0.92
4	16.4	0.94	17.0	0.94
*5*	*18.2*	*0.96*	*19.8*	0.97
10	18.8	0.95	21.4	0.97
15	19.2	0.92	22.2	0.97
20	17.6	0.92	21.4	0.98

The start-time of 5 min (values shown in italics) was selected with the MA1 V_T_ value closest to the 1TC model reference value of 20 mL/cm^3^ and the highest Logan r^2^

### PET image analysis

**Fig. 2:**
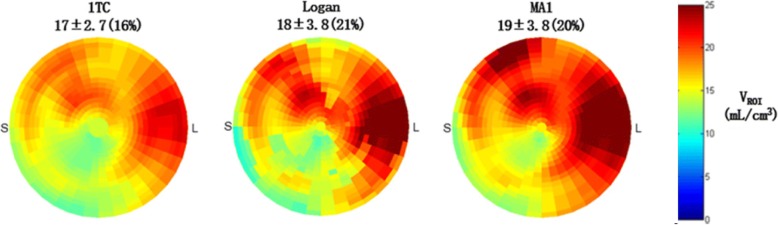
Example polar-maps of V_ROI_ estimated from a single patient scan using the one-tissue-compartment (1TC), Logan, and MA1 models. The polar-maps demonstrate similar regional patterns and global median values

**Fig. 3:**
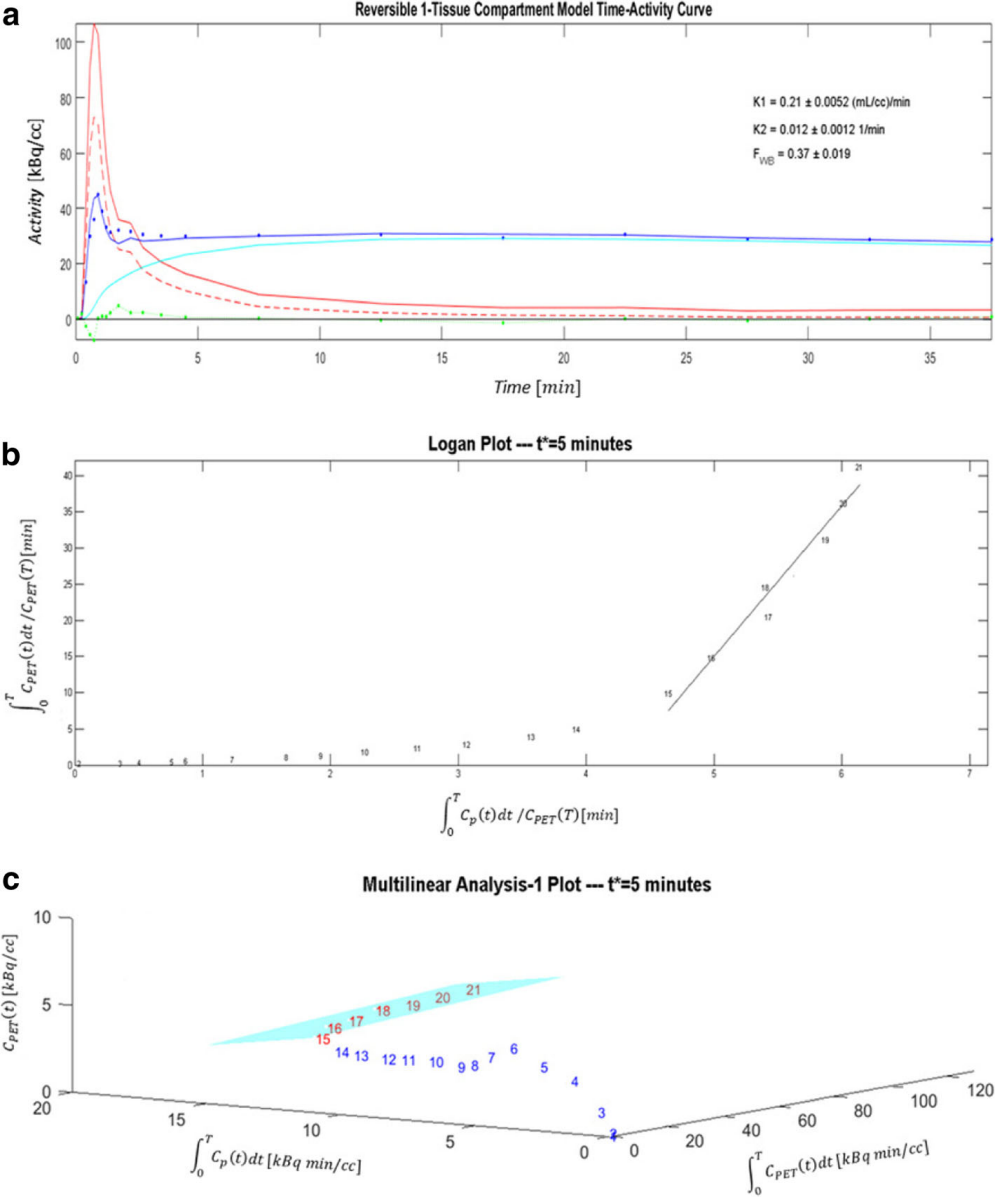
Model fitting results for the same patient scan shown in Fig. 2. **a** One-tissue-compartment (1TC) analysis of [^11^C]HED PET data showing time-activity curves in arterial whole-blood (red) and metabolite-corrected arterial plasma (dotted red), as well as LV myocardial ROI (dark blue) and myocardial tissue alone (cyan). Residuals (measured–modeled PET data) are shown in green. **b** Logan and **c** MA1 plots of LV myocardial uptake demonstrating that steady-state (linear response) is reached after approximately 5 min post-injection (frame #15)

Figure 2 shows V_T_ polar-maps from a single patient scan using all 3 models, including graphical representations of the Logan and MA1 plots as well as the 1TC modeling results in Fig. 3. V_T_ polar-maps were found to show very similar spatial distributions for all three kinetic models, as expected.

### Comparison of Logan and MA1 versus 1TC

At baseline, V_T_ values were 20 ± 8 mL/cm^3^ for 1TC, 17 ± 8.0 mL/cm^3^ for Logan, and 20 ± 16 mL/cm^3^ for MA1, as shown in Table 3. At follow-up, V_T_ values were 21 ± 11 mL/cm^3^ for 1TC, 19 ± 12 mL/cm^3^ for Logan, and 23 ± 16 mL/cm^3^ for MA1. Intra-model comparison of the V_T_ values at baseline vs. follow-up revealed that there was no significant difference between baseline and follow-up V_T_ values for any of the models (*p* = 0.379). However, on average, V_T_ values generated by Logan were 15% lower than those generated by MA1 (*p* = 0.002) and 12% lower than the 1TC values (*p* = 0.002). V_T_ values generated by MA1 were not significantly different from those of 1TC (*p* = 0.958).

**Table 3:** [^11^C]HED PET V_T_ Measurements (*N* = 18)

Parameter	1TC		Logan*		MA1	
	Baseline	Follow-up	Baseline	Follow-up	Baseline	Follow-up
V_T_	19.7 ± 7.8	21.3 ± 11.5	17.3 ± 8.0	18.8 ± 12.0	20.1 ± 9.5	22.6 ± 16.2

*Significant differences between Logan vs 1TC (*p* = 0.002) and Logan vs MA1 (p = 0.002)

**Fig. 4:**
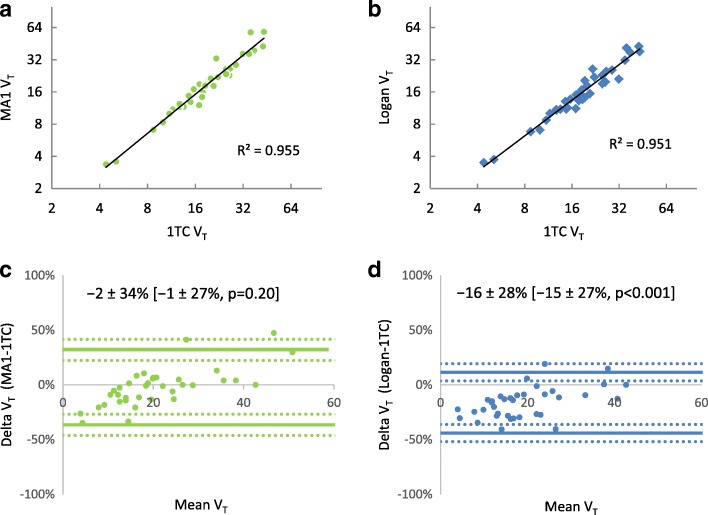
Inter-model reproducibility of [^11^C]HED PET measurements of V_T_ using MA1 (**a**, **b)** and Logan (**c**, **d**) models. Scatter-plots (**a**, **c**) show excellent correlation of the graphical model values versus the 1TC standard with *R*^2^ > 0.95. Bland Altman plots (**b**, **d**) show the 95% limits-of-agreement (dotted lines) and confidence intervals (shaded areas) at Delta = mean ± 1.96 × SD. [Values] are Delta = median ± 1.45 × IQR, *p* value assessed by the Wilcoxon Signed Rank Test

To evaluate reproducibility between the three models, the 1TC model was taken as the reference standard for comparison with the Logan and MA1 models. Of the 36 scans compared in the inter-model analysis (Table 4, Fig. 4), the Logan-vs-1TC and MA1-vs-1TC comparisons exhibited similar reproducibility with NPC ~ 26.5%. However, the V_T_ values generated from the Logan model were systematically lower than those generated from the 1TC model (median bias = − 14.5% and mean bias = − 16.3%, *p* < 0.001), but there was no systematic difference in V_T_ when comparing MA1-vs-1TC models, *p* = 0.2). There was excellent agreement between MA1-vs-1TC values (ICC = 0.955, 95% CI [0.915, 0.977]) and good-to-excellent agreement between Logan-vs-1TC (ICC = 0.928, 95% CI [0.432, 0.978]). There was no difference in reproducibility between the MA1-vs-1TC and Logan-vs-1TC NPC values (nonparametric Levene’s test, *p* = 0.915).

**Table 4:** Inter-model reproducibility of V_T_ measurements (*N* = 36)

Models compared	ICC [95%CI]	Average Delta ± RPC	Median Delta ± NPC
Logan vs 1TC	0.928 [0.432, 0.978]	− 16.3 ± 27.8% †	− 14.5 ± 26.6%^†^
MA1 vs 1TC	0.955 [0.915, 0.977]	− 2.3 ± 34.2%	− 1.0 ± 26.5%

^†^Significant bias vs zero (*p* < 0.001)

### Test-retest repeatability of kinetic models

**Fig. 5:**
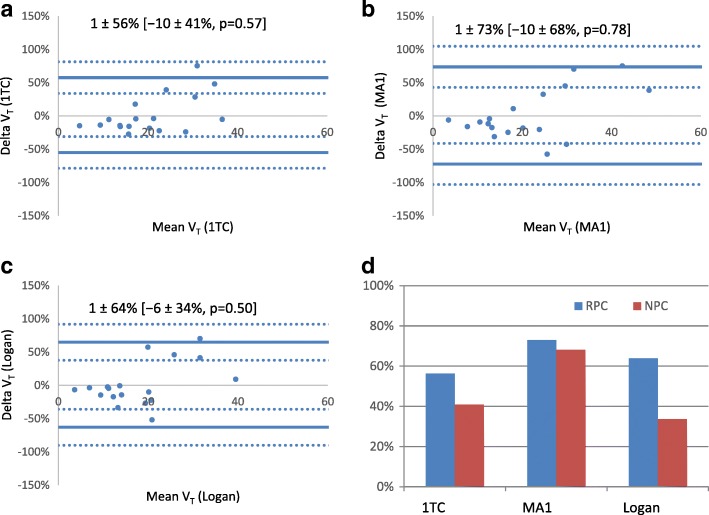
Test-retest repeatability of [^11^C]HED PET measurements (*N* = 18 scans) for 1TC (**a**), MA1 (**b**), and Logan (**c**) methods. 95% limits-of-agreement (solid lines) and their confidence intervals (dotted lines) are shown at Delta = mean ± 1.96 × SD. [Values] are Delta = median ± 1.45 × IQR, *p* value assessed by the Wilcoxon Signed Rank Test. Average RPC and NPC values in **d** are 64 and 48%. *F*-test showed no differences in the baseline-follow-up variability between the three models (*p* < 0.05)

All models demonstrated very good repeatability (Table 5) with consistent ICC values = 0.837–0.852. The mean test-retest differences were all < 2% without any systematic bias observed between baseline and follow-up (Fig. 5), but this could be the result of relatively small sample size with fewer points (*N* = 18) compared to the inter-method analysis (*N* = 36). There was no difference in the test-retest reproducibility (NPC) values between the three methods (non-parametric Levene’s test, *p* = 0.57).

**Table 5:** Test-retest repeatability of V_T_ measurements (*N* = 18)

Model	ICC [95%CI]	Average Delta ± RPC	Median Delta ± NPC
1TC	0.852 [0.645, 0.942]	1.3 ± 56.2%	− 9.6 ± 40.9%
Logan	0.852 [0.646, 0.942]	0.8 ± 73.0%	− 10.4 ± 68.1%
MA1	0.837 [0.614, 0.936]	0.9 ± 63.9%	− 5.5 ± 33.7%

## Discussion

In an effort to improve and expand the use of kinetic modeling in cardiac PET studies of sympathetic innervation, we sought to evaluate multiple kinetic models for the analysis of [^11^C]HED studies. This was achieved by comparing the inter-method differences in V_T_ quantified by the Logan and MA1 graphical models compared to the reference 1TC model in a sample of heart failure patients and assessing the test-retest repeatability between baseline and follow-up scans. HED PET is often used to evaluate therapy or disease progression in heart failure patients; therefore, evaluation of the test-retest repeatability is most relevant in this same population, as opposed to healthy normal subjects who generally have lower sympathetic tone. The patients’ heart failure symptoms and medications were stable over the test-retest interval; therefore, any impact on the repeatability data should be minimal.

The MA1 model exhibited excellent agreement with 1TC, the Logan model exhibited good-to-excellent agreement with 1TC, and all models had good-to-excellent test-retest repeatability. Logan V_T_ values were significantly lower than MA1 and 1TC V_T_ values, while MA1 V_T_ values were not significantly different from those obtained using the 1TC model (Table 3). While 1TC is the reference standard kinetic model in this instance, graphical models such as the Logan and MA1 are computationally simpler alternatives that allow for linearized visualization and analysis of tracer kinetic data. Our findings support the reliable use of both graphical analysis methods in addition to the standard 1TC model for tracer kinetic analysis of V_T_. These findings agree with previous studies using other PET tracers that compared various graphical models, including the Logan method, finding the results to be in agreement with standard compartment models, but computationally simpler, and potentially more robust [24–27].

In the present cardiac PET study, partial volume and spillover corrections were critical to implement into the graphical modeling calculations to avoid misinterpretation. The commonly used Logan and MA1 methods (Eqs. 12 and 13) only estimate the volume of distribution in the PET image region (V_ROI_) as opposed to the myocardial tissue of interest (V_T_). Compared to PET measurements in other organ systems such as the brain, in cardiac studies, the measured ROI region contains much more spillover of blood signal within and adjacent to the myocardial tissues. Our implementation of a partial-volume correction method based on estimated recovery coefficients and whole-blood spillover fractions allowed accurate measurement of myocardial V_T_ values using Logan and MA1 graphical models on a scan-specific basis. In this validation study, F_WB_ was estimated first using the 1TC with spillover model, and then used to calculate the corresponding RC values for consistent partial-volume and spillover correction of the graphical model V_ROI_ estimates. It is clear that independent estimates of RC and F_WB_ are required to determine V_T_ from V_ROI_ as shown in Eq. 11; therefore, any error in the estimation of these correction factors in practice will be propagated directly into the corresponding values of V_T_. In the present study, the average F_WB_ value was 0.37 ± 0.07, which could be used to estimate RC and hence V_T_ in similar patient population studies with minimal added variability.

We investigated the effect of varying t* on the graphical model results (Table 2), which quantified V_T_ using the plotted values at *t* ≥ t*. It has been reported that t* may be deduced directly from kinetic modeling data for some tracers [5], but the method we presented used a simpler and systematic approach to determine the t* which produced the same V_T_ values on average compared to the MA1 plots. This approach is beneficial for tracers for which it is more difficult to estimate t* directly from the study data, such as those with relatively slower kinetics [28]. It also removes the need to estimate t* for each individual scan, which may be subject to variable noise effects. We propose t* = 5 min as an effective start-time for cardiac studies employing [^11^C]HED as it also gave the highest quality of linear fit (*r*^2^ > 0.95) using the Logan model, in addition to MA1 estimates of V_T_ that were equal to the 1TC reference value on average. This start time was shown with our comparison of the three models to be robust, producing results for V_T_ with excellent goodness-of-fit to the graphical models and inter-method agreement. It is worth noting that a slightly later start time of 10–15 min may have provided Logan V_T_ values that correspond better with 1TC and MA1 (Fig. 1), but at the cost of a lower quality fit of the linear model and wider variability due to fewer fitted points.

Interestingly, the V_T_ values determined by Logan were significantly lower than those determined by both MA1 and 1TC, while V_T_ values determined by MA1 did not show a significant difference to those obtained from 1TC. More precisely, Logan exhibited a greater negative bias where V_T_ was underestimated relative to 1TC, whereas a bias was not present between MA1 and 1TC (Table 4). In a similar kinetic model comparison using [^18^F]FCWAY and [^11^C]MDL neurological tracers, Ichise et al. [6] demonstrated that the MA1 model generated higher V_T_ estimates than Logan, and that MA1 exhibited less bias compared to Logan at multiple imaging noise levels. Our results are consistent with these findings, affirming the original report of MA1 as a method to reduce the magnitude of bias induced by noise when using the Logan model [6]. Although Logan seemed to underestimate V_T_ in our study population, it should be realized that the median bias of − 14.5% relative to the 1TC gold standard did not greatly affect the inter-model reproducibility of the models, which exhibited good to excellent agreement despite the bias that was present.

The use of [^11^C]HED to examine sympathetic function in cardiac PET is becoming increasingly widespread. Recently, it has been shown to be a powerful diagnostic and prognostic tool for patients with heart failure, arrhythmias, flow-innervation mismatches, and microvascular dysfunction in both infarcted and non-infarcted tissues [1, 29–33]. This field continues to be improved and shows promise for a wider variety of applications [34]. As cardiac innervation tracers increase in prevalence, the optimization and validation of kinetic modeling techniques becomes more important; extensions of the current study may be anticipated, such as those investigating the use of a two-tissue-compartment model to quantify cardiac NET re-uptake function more specifically. Moreover, comparisons of multiple kinetic modeling options, in particular those of a graphical nature as presented here, are possible with other cardiac innervation-based tracers such as the [^18^F]-labeled sympathetic innervation tracers MFBG, MHPG, LMI1195, etc., for more detailed evaluation of their kinetics [35, 36].

A few limitations were present in this study. The current study is a retrospective, single-center study that examined stable heart failure patients only from the PET-OSA trial. The results may be limited by the relatively small sample size (*N* = 18). Larger prospective studies would be beneficial to further validate the performance of the kinetic models as proposed.

## Conclusion

A start time of 5 min was found to provide the best fit for Logan and MA1 models. The MA1-1TC comparison demonstrated excellent agreement while Logan-1TC and test-retest comparisons demonstrated good-to-excellent agreement when quantifying V_T_ with partial volume correction. Although Logan underestimated V_T_ due to the recognized noise bias, Logan and MA1 both exhibited similar test-retest variability, suggesting that they may be used in addition to 1TC in the modeling of [^11^C]HED kinetics, with benefits of greater computational simplicity and the ability to mathematically visualize kinetic parameters for better quality assurance.

## Additional file

Additional file 1: **Figure S1.** Unchanged parent fraction in plasma (cyan) is calculated as the product of the plasma-to-whole blood fraction (green) times the unchanged parent fraction (blue) curves, derived from the human data presented in Harms et al. [3]. (DOCX 61 kb)
