# Supplementary material for: Reproducible quantification of cardiac sympathetic innervation using graphical modeling of carbon-11-meta-hydroxyephedrine kinetics with dynamic PET-CT imaging
Source: EJNMMI Res. 2018 Jul 20;8:63. doi: 10.1186/s13550-018-0421-5 (PMC6054601; doi:10.1186/s13550-018-0421-5)
Supplement: Supplementary file 1 — Figure S1. Unchanged parent fraction in plasma (cyan) is calculated as the product of the plasma-to-whole blood fraction (green) times the unchanged parent fraction (blue) curves, derived from the human data presented in Harms et al. [3]. (DOCX 61 kb) [file 13550_2018_421_MOESM1_ESM.docx]

**Figure S1.** Unchanged parent fraction in plasma (cyan) is calculated as the product of the plasma-to-whole blood fraction (green) times the unchanged parent fraction (blue) curves, derived from the human data presented in Harms et al [3].


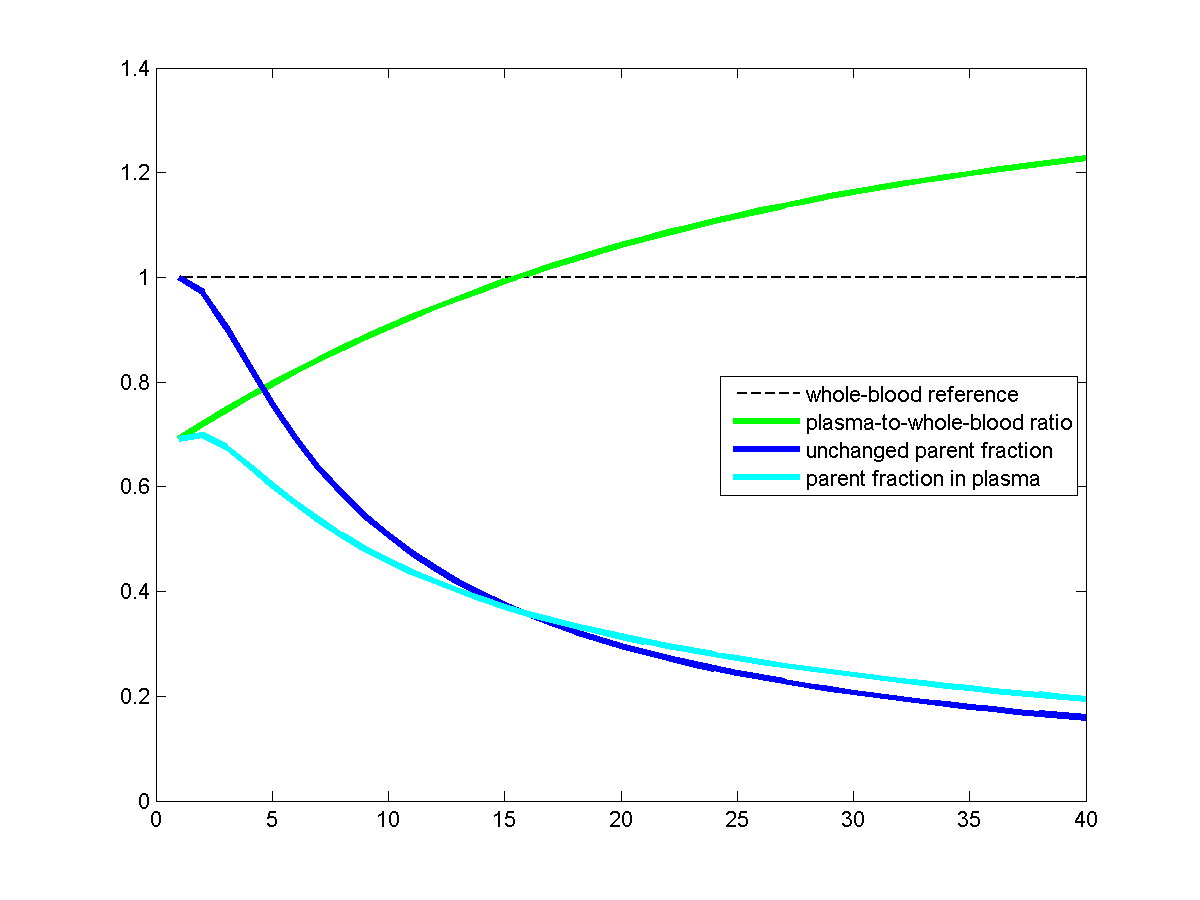


Time [minutes]

Fraction [unitless]
